# The Profile of Microbiological Pathogens in Diabetic Foot Ulcers

**DOI:** 10.3389/fmed.2021.656467

**Published:** 2021-09-21

**Authors:** Wei Chai, Yuqing Wang, Huancheng Zheng, Song Yue, Yan Liu, Yili Wu, Xuezhi Li

**Affiliations:** ^1^Department of Surgery, Tianjin Second Hospital, Tianjin, China; ^2^Shandong Collaborative Innovation Center for Diagnosis, Treatment and Behavioral Interventions of Mental Disorders, Institute of Mental Health, Jining Medical University, Jining, China; ^3^Shandong Key Laboratory of Behavioral Medicine, School of Mental Health, Jining Medical University, Jining, China; ^4^Cheeloo College of Medicine, Shandong University, Jinan, China; ^5^Key Laboratory of Alzheimer's Disease of Zhejiang Province, School of Mental Health and The Affiliated Kangning Hospital, Institute of Aging, Wenzhou Medical University, Wenzhou, China; ^6^Oujiang Laboratory, Wenzhou, China

**Keywords:** diabetic foot ulcer, infection, pathogens, antibiotic, susceptibility

## Abstract

Diabetic foot, a main complication of diabetes mellitus, renders the foot susceptible to infection, and may eventually lead to non-traumatic limb amputation due to the deterioration of diabetic foot ulcers (DFUs). Characterizing the pathogen spectrum and antibiotic susceptibility is critical for the effective treatment of DFUs. In the current study, the characteristics and antibiotic susceptibility of the pathogen spectrum were analyzed. Secretions from the DFUs of 102 patients were cultured, and dominant pathogens were identified by using test cards. Antibiotic susceptibility of dominant pathogens was assayed by the Kirby–Bauer assay. We found that the dominant pathogens varied with age, duration of diabetes, blood sugar control, and the initial cause of ulcers. Moreover, the dominant pathogens were susceptible to at least one antibiotic. However, the antibacterial efficacy of several commonly used antibiotics decreased from 2016 to 2019. Our study indicates that the identification of dominant pathogens and antibiotic susceptibility testing is essential for the treatment of DFUs with effective antibiotics, while the abuse of antibiotics should be strictly restrained to reduce the generation of antibiotic-resistant strains.

## Introduction

Diabetes mellitus (DM) is one of the most common chronic diseases. According to an International Diabetes Federation report, globally one in 11 adults aged 20–79 years (~463 million people) were living with diabetes in 2019, and approximately a quarter of those were in China. A main complication of DM is a diabetic foot, which renders the foot susceptible to infection and can eventually lead to non-traumatic limb amputation. Diabetic foot infection (DFI) is defined as the infection of tissue below the ankle in people with diabetes (1, 2). DM patients with deep foot infections are 154.5 times more likely to have a leg, foot, or toe amputated compared with patients without DM (3, 4). The infection is usually complicated to diabetic foot ulcer (DFU) initiated by an open wound on the foot caused by injury, ischemic, or tinea pedis. The weakened immune system, impaired peripheral blood circulation, neuropathy, and peripheral vasculopathy facilitate DFI (2, 5, 6).

The pathogens of DFI include aerobic bacteria such as *Staphylococcus, Streptococcus*, and *Enterobacteriaceae*, and anaerobic bacteria such as *Bacteroides, Clostridium*, and *Peptostreptococcus*, and fungi (7–9). According to guidelines compiled by the Infectious Diseases Society of America (10, 11), DFIs are classified into three subcategories, namely mild infections with only superficial symptoms that are limited in size and depth, moderate infections with deeper or more extensive symptoms, and severe infections accompanied by systemic signs or metabolic perturbations (10, 11). For the treatment of mild and moderate DFIs, oral therapy alone or followed by a short course of intravenous therapy with narrow-spectrum antibiotics is likely sufficient. Severe DFI is often associated with previously treated chronic infection, and possibly with antibiotic resistance. The initial approach for severe DFI treatment is parenteral administration of broad-spectrum antibiotics, minimally those against *Staphylococcus* and *Streptococcus* (12, 13). However, optimal approaches for DFI treatment require additional clinical data. Thus, characterizing the pathogen spectrum and antibiotic susceptibility is critical for the effective treatment of DFUs. The current study was designed to analyze the pathogen spectrum and antibiotic susceptibility in patients with DFUs. The results may provide new information for optimizing definitive therapy for DFUs.

## Materials and Methods

### Subjects

Patients with DFUs admitted to the Department of Diabetic Foot at Tianjin Second People's Hospital were recruited from 2016 to 2019. All the diagnoses of the patients were made based on clinical and laboratory examinations. The medical records and collected demographic data, including age, gender, initial cause of ulcers, diabetes duration, and glycemic control were reviewed. Inclusion criteria were as follows: (1) DFUs were at Grades III or above according to the Wagner DFU grade classification system; (2) dominant pathogens were detected. The dominant pathogen was defined as the bacteria strain accounting for more than 50% of the total bacteria strains identified from a single secretion sample from DFUs. Exclusion criteria included the following: (1) DFUs were at Grades I or II according to the Wagner DFU Grade Classification System; (2) no dominant pathogen was detected; (3) patients with other diseases accompanied by DFUs were not admitted to the Department of Diabetic Foot. Informed consent was obtained from the patients, and the study was approved by the Ethical Committee of Jining Medical University (#2021-YX-ZR-009).

### Pathogen Identification

On the day of admission, the ulcerated secretions were collected using traditional ulcer swabs and cultured within 1 h after collection. Collected secretions were cultured in blood–agar plate at 35°C for 24 h. Pathogen spectrum was identified using the test cards of VITEK-60 automated microbial identification systems from bioMerieumx (Marcy-l'Étoile, France).

### Antibiotic Susceptibility Test

Antibiotic susceptibility test was carried out by Kirby–Bauer assay. Quality control strains include *Staphylococcus aureus* (ATCC25923), *Escherichia coli* (ATCC25922) and *Pseudomonas aeruginosa* (ATCC27853). After overnight incubation on Mueller-Hinton agar plate, the zone sizes (area of no growth around the disk) were measured, and the minimum inhibitory concentration (MIC) was calculated based on the zone sizes. The results were interpreted as resistant (R), intermediate (I), or sensitive (S) for each antimicrobial according to the ranges recommended by the China Antimicrobial Resistance Surveillance System. For the therapeutic outcomes of the patients, the cure rate was the percentage of patients with complete healing.

## Results

### General Patient Information

Of the total of 102 patients, 37 (36.3%) were female and 65 (63.7%) were male. There were 1.76 times more male patients than female patients. The average age was 72.42 ± 8.43 ($\bar{x}$ ± SD) years, and 45.1% of the patients were aged between 71 and 80 years. The average diabetes duration was 10.09 ± 3.40 years, and 63.7% of the patients had a DM history of 6–10 years. Glycemic control was poor (fasting glucose > 10 mmol/L) in 66.7 % of the patients. With regard to the pathogeny of DFI, 63.7% of the cases were caused by injury, 18.6% were caused by ischemia, and 17.6% were caused by tinea pedis. Demographic and clinical characteristics of the patients are shown in Table 1.

**Table 1:** General information of the 102 DFI patients.

**Index**	**Categories**	**Numbers (%)**
Sex	Female	37 (36.3)
	Male	65 (63.7)
Age	≤60	10 (9.8)
	61–70	33 (32.4)
	71–80	46 (45.1)
	≥81	13 (12.7)
Years with DM	≤5	4 (3.9)
	6–10	65 (63.7)
	11–15	23 (22.5)
	16–20	9 (8.8)
	≥21	1 (1.0)
Glycemic control	poor	68 (66.7)
	Well	34 (33.3)
Initial cause of ulcers	Injury	65 (63.7)
	Tinea pedis	18 (17.6)
	Ischemic rupture	19 (18.6)

### Dominant Pathogen

The dominant pathogens were identified from the ulcer secretions of 102 patients. Detailed information pertaining to the pathogens is presented in Table 2. Of all the pathogens identified, 54.9% (*n* = 56) were Gram-negative bacteria and 43.1% (*n* = 44) were Gram-positive bacteria. On the other hand, 53.9% (*n* = 55) of the bacteria were pathogenic bacteria and 44.1% (*n* = 45) were conditional pathogenic bacteria that were only pathogenic under certain conditions, such as wounds and a decrease of immune function. Fungal infections were only detected in two patients (2.0%).

**Table 2:** Identified pathogens from the secretions of the 102 DFI patients.

**Strain type**	**2016**		**2017**		**2018**		**2019**		**Total**	
	***n***	**%**	***n***	**%**	***n***	**%**	***n***	**%**	***n***	**%**
• *Gram negative bacillus*	12	50.0	14	53.8	15	57.7	15	57.7	56	54.9
*^1^Pseudomonas aeruginosa*	4	16.7	4	15.4	6	23.1	6	23.1	20	19.6
^1^ *Klebsiella pneumoniae*	3	12.5	1	3.8	2	7.7	2	7.7	8	7.8
^2^ *Escherichia coli*	3	12.5	5	19.2	3	11.5	4	15.4	15	14.7
^2^ *Citrobacter*	1	4.2	1	3.8	2	7.7	1	3.8	5	4.9
^2^ *Morganella morganii*	0	/	1	3.8	0	/	0	/	1	1.0
^2^ *Enterobacter aerogenes*	0	/	0	/	0	/	0	/	0	/
^2^ *Acinetobacter lwoffii*	0	/	0	/	0	/	0	/	0	/
^2^ *Acinetobacter calcoaceticus*	0	/	0	/	0	/	0	/	0	/
^2^ *Enterobacter cloacae*	1	4.2	2	7.7	2	7.7	2	7.7	7	6.9
• *Gram-positive bacterium*	11	45.8	12	46.2	11	42.3	10	38.5	44	43.1
^2^ *Enterococcus faecalis*	4	16.7	4	15.4	2	7.7	2	7.7	12	11.8
^1^ *Staphylococcus aureus*	4	16.7	5	19.2	5	19.2	5	19.2	19	18.6
^1^ *Streptococcus haemolyticus*	2	8.3	2	7.7	2	7.7	2	7.7	8	7.8
^2^ *Staphylococcus epidermidis*	1	4.2	1	3.8	2	7.7	1	3.8	5	4.9
*Others*	0	/	0	/	0	/	0	/	0	/
• *Fungal*	1	4.2	0	/	0	/	1	3.8	2	2.0
*Candida albicans*	1	4.2	0	/	0	/	1	3.8	2	2.0
*Candida tropicalis*	0	/	0	/	0	/	0	/	0	/
*Others*	0	/	0	/	0	/	0	/	0	/
Total	24	100.0	26	100.0	26	100.0	26	100	102	100.0
1: pathogenic bacteria	13	54.2	12	46.2	15	57.7	15	57.7	55	53.9
2: conditional pathogen bacteria	10	41.7	14	53.8	11	42.3	10	38.5	45	44.1

To further investigate the distribution of pathogens, the patients were divided into groups based on sex, age, DM duration, glycemic control, and initial cause of ulcers. The pathogen spectrums in the different patient groups are shown in Supplementary Table 1. The specific distributions of the pathogens identified inside the different patient groups are shown in Supplementary Table 2. Up to 87.5% of the *Streptococcus haemolyticus* infections and 83.3% of the *Enterococcus faecalis* infections were identified in male patients. Conversely, 80% of the *Citrobacter* infected patients were female, even though there were far fewer female patients in the study than male patients. More than 70% of *E. faecalis* (83.3%), *E. coli* (80.0%), *Citrobacter* (80.0%), *S. haemolyticus* (75.0%), and *S. aureus* (73.7%) infections were detected in patients with DM histories ranging from 6 to 10 years, and more than 70% of *E. faecalis* (91.7%), *Staphylococcus epidermidis* (80%), *Citrobacter* (80%), *S. aureus* (78.9%), and *Enterobacter cloacae* (71.4%) infections were detected in patients with poor blood control (fasting glucose > 10 mmol/L). With regard to the initial causes of ulcers, more than 80% of *S. aureus* (100%), *E. coli* (93.3%), and *E. faecalis* (83.3%) infections were detected in the injury group.

### Antibiotic Susceptibility

For drug susceptibility testing, 56 selected bacterial isolates were assigned to *Staphylococcus* (*n* = 24), *Enterococcus* (*n* = 11), and *Acinetobacter* and *Pseudomonas* (*n* = 20) pathogen groups based on their genus. A total of 16 commonly used antibiotics were used to analyze antibiotic susceptibility in these three groups (Tables 3–**5**).

**Table 3:** Susceptibility of *Staphylococcus* pathogens to antibiotics.

***Antibiotics***	**2016 (** ***n*** **= 5)**		**2017 (** ***n*** **= 6)**		**2018 (** ***n*** **= 7)**		**2019 (** ***n*** **= 6)**		**Total (** ***n*** **= 24)**	
	***n***	**%**	***n***	**%**	***n***	**%**	***n***	**%**	***n***	**%**
*Cefazolin*	0	/	0	/	0	/	0	/	0	/
*Ceftazidime*	0	/	0	/	0	/	0	/	0	/
*Cefoperazone*	0	/	0	/	0	/	0	/	0	/
*Cefodizime*	0	/	0	/	0	/	0	/	0	/
*Gentamicin*	0	/	0	/	0	/	0	/	0	/
*Ciprofloxacin*	3	60.0	2	33.3	0	/	0	/	5	20.8
*Levofloxacin*	4	80.0	4	66.7	0	/	3	50.0	11	45.8
*Imipenem*	4	80.0	3	50.0	3	42.8	4	66.7	14	58.3
*Tetracycline*	1	20.0	0	/	4	57.1	3	50.0	8	33.3
*Chloramphenicol*	3	60.0	2	33.3	0	/	4	66.7	9	37.5
*Piperacillin*	0	/	0	/	0	/	0		0	/
*Vancomycin*	5	100.0	6	100.0	7	100.0	6	100.0	24	100.0
*Clindamycin*	0	/	0	/	0	/	0	/	0	/
*Amikacin*	0	/	0	/	0	/	0	/	0	/
*Meropenem*	4	80.0	5	83.3	5	71.4	4	66.7	18	75.0
*Rifampicin*	4	80.0	3	50.0	6	85.7	0	/	13	54.2

With respect to *Staphylococcus*, all the 24 strains were susceptible to vancomycin, whereas susceptibility to another seven antibiotics tested was variable from 2016 to 2019. Susceptibility to ciprofloxacin was decreased from 2016 to 2017, and no ciprofloxacin susceptibility was detected in 2018 or 2019. No susceptibility to gentamicin, piperacillin, clindamycin, amikacin, or the four cephalosporins was detected (Table 3).

The 11 *Enterococcus* strains exhibited substantial variation in antibiotic susceptibility, possibly because of the comparatively smaller number of strains. Four antibiotics were ineffective in all strains, and three antibiotics had effects in one of the 11 strains. Levofloxacin, chloramphenicol, cefazolin, and cefodizime were effective in 2016 and 2017 but not in 2018 and 2019. Imipenem was effective in 2016 and 2019. Vancomycin, meropenem, and rifampicin were 100% effective from 2016 to 2019 (Table 4).

**Table 4:** Susceptibility of *Enterococcus* pathogens to antibiotics.

***Antibiotics***	**2016 (** ***n*** **= 3)**		**2017 (** ***n*** **= 4)**		**2018 (** ***n*** **= 2)**		**2019 (** ***n*** **= 2)**		**Total (** ***n*** **= 11)**	
	***n***	**%**	***n***	**%**	***n***	**%**	***n***	**%**	***n***	**%**
*Cefazolin*	2	66.7	1	25.0	0	/	0	/	3	27.3
*Ceftazidime*	0	/	0	/	0	/	0	/	0	/
*Cefoperazone*	1	33.3	0	/	0	/	0	/	1	9.1
*Cefodizime*	2	66.7	3	75.0	0	/	0	/	5	45.5
*Gentamicin*	0	/	0	/	0	/	1	50.0	1	9.1
*Ciprofloxacin*	1	33.3	0	/	0	/	0	/	1	9.1
*Levofloxacin*	3	100.0	0	/	0	/	0	/	3	27.3
*Imipenem*	3	100.0	0	/	0	/	2	100.0	5	45.5
*Tetracycline*	3	100.0	0	/	2	100.0	0	/	5	45.5
*Chloramphenicol*	3	100.0	0	/	0	/	0	/	3	27.3
*Piperacillin*	0	/	0	/	0	/	0	/	0	/
*Vancomycin*	3	100.0	4	100.0	2	100.0	2	100.0	11	100.0
*Clindamycin*	0	/	0	/	0	/	0	/	0	/
*Amikacin*	0	/	0	/	0	/	0	/	0	/
*Meropenem*	3	100.0	4	100.0	2	100.0	2	100.0	11	100.0
*Rifampicin*	3	100.0	4	100.0	2	100.0	2	100.0	11	100.0

For the 20 *Acinetobacter* and *Pseudomonas* strains, four antibiotics were ineffective in all strains, and six antibiotics were occasionally effective. Imipenem was effective in 40.0% of strains, and tetracycline was effective in 30.0% of strains. Chloramphenicol was effective in 75% strains isolated in 2016, but was completely ineffective in the 17 strains isolated from 2017 to 2019. Vancomycin was 100% effective in all the 20 strains isolated from 2016 to 2019. Meropenem was effective in all the four strains isolated in 2016, but its effectiveness was decreased to 66.7% by 2019. Only vancomycin and rifampicin were effective in all isolates throughout the study (2016–2019) (Table 5).

**Table 5:** Susceptibility of *Acinetobacter and Pseudomonas* pathogens to antibiotics.

***Antibiotics***	**2016 (** ***n*** **= 4)**		**2017 (** ***n*** **= 4)**		**2018 (** ***n*** **= 6)**		**2019 (** ***n*** **= 6)**		**Total (** ***n*** **= 20)**	
	***n***	**%**	***n***	**%**	***n***	**%**	***n***	**%**	***n***	**%**
*Cefazolin*	1	25.0	0	/	0	/	0	/	1	5.0
*Ceftazidime*	0	/	0	/	0	/	0	/	0	/
*Cefoperazone*	0	/	0	/	0	/	0	/	0	/
*Cefodizime*	2	50.0	0	/	0	/	0	/	2	10.0
*Gentamicin*	2	50.0	0	/	0	/	0	/	2	10.0
*Ciprofloxacin*	0	/	0	/	0	/	1	16.7	1	5.0
*Levofloxacin*	0	/	0	/	2	33.3	0	/	2	10.0
*Imipenem*	0	/	2	50.0	3	50.0	3	50.0	8	40.0
*Tetracycline*	0	/	0	/	3	50.0	3	50.0	6	30.0
*Chloramphenicol*	3	75.0	0	/	0	/	0	/	3	15.0
*Piperacillin*	0	/	0	/	1	16.7	0	/	1	5.0
*Vancomycin*	4	100.0	4	100.0	6	100.0	6	100.0	20	100.0
*Clindamycin*	0	/	0	/	0	/	0	/	0	/
*Amikacin*	0	/	0	/	0	/	0	/	0	/
*Meropenem*	4	100.0	3	75.0	4	66.7	4	66.7	15	75.0
*Rifampicin*	4	100.0	4	100.0	6	100.0	6	100.0	20	100.0

### Therapeutic Outcomes

All 102 DFI patients were treated with surgical dressing changes, circulation improvement, and antibiotics based on microbiological examination and drug sensitivity test results. The therapeutic outcomes are shown in Table 6. The standard of cure is complete recovery and ulcer healing. The standard of improvement is the ulcer not completely healed, but requiring outpatient dressing change treatment and home rest. Amputation types include toe amputation, hemipod amputation, mid-upper third of the leg amputation, mid-lower third of the thigh amputation, and mid-upper third of the thigh amputation. The six amputation cases in our study included three cases of mid-lower third of the thigh amputation, two cases of mid-upper third of the leg amputation, and one case of hemipod amputation. Infections with common strains such as *S. aureus, E. faecalis, E. coli*, and *P. aeruginosa* had better therapeutic outcomes, with cure rates ≥ 70%. In contrast, infections with some rare strains including *S. epidermidis, Citrobacter*, and *Klebsiella pneumoniae* had lower cure rates. The two fungal infections detected in the study were completely cured.

**Table 6:** Therapeutic outcomes of the 102 DFI patients.

**Infected strain type**	**Cure**		**Improve**		**Amputation**		**Total**
	***n***	**%**	***n***	**%**	***n***	**%**	
• ***Gram-negative bacteria***	42	75.0	10	17.9	4	7.1	56
*Pseudomonas aeruginosa*	14	70.0	3	15.0	3	15.0	20
*Klebsiella pneumoniae*	5	62.5	2	25	1	12.5	8
*Escherichia coli*	14	93.3	1	6.7	0	/	15
*Citrobacter*	3	60.0	2	40.0	0	/	5
*Morganella morganii*	1	100.0	0	/	0	/	1
*Enterobacter cloacae*	5	71.4	2	28.6	0	/	7
• ***Gram-positive bacteria***	39	88.6	3	6.8	2	4.5	44
*Enterococcus faecalis*	11	91.7	0	/	1	8.3	12
*Staphylococcus aureus*	19	100.0	0	/	0	/	19
*Streptococcus haemolyticus*	7	87.5	0	/	1	12.5	8
*Staphylococcus epidermidis*	2	40.0	3	60.0	0	/	5
• ***Fungal***	2	100.0	0	/	0	/	2
*Candida albicans*	2	100.0	0	/	0	/	2
Total	83	81.4	13	12.7	6	5.9	102

## Discussion

Pathogenic bacteria and drug sensitivity spectrums vary regionally and are affected by the widespread use of antibiotics. Appropriate antibiotic selection for DFI is controversial because to date no empirical antimicrobial regimen has been shown to be superior. Thus, definitive therapy should be based on the identification of pathogens and their drug sensitivity. The current study generated drug susceptibility results for a variety of bacterial pathogens isolated from patients with DFI. All of the clinical profiles were derived from inpatients and outpatients living in urban areas of Tianjin, China. Thus, caution must be observed while interpreting the results.

The baseline characteristics of the sample population are consistent with those previously reported in European DFI studies (14, 15), in which 63.7% of the patients were male and 66.7% of the patients had poor glycemic control. The vast majority of the patients (90.2%) were older than 61 years and 96.1% of the patients had a >6 year history of DM. Injury was the main initial cause of DFI, accounting for 63.7% of all cases. These findings are consistent with previous reports in which male gender and poor glycemic control were independent risk factors for infection and non-healing DFIs (16). The predominance of males may be due to the fact that they are more commonly exposed to the outside environment compared with females (17).

With regard to bacterial distribution, a relatively large number of Gram-negative bacteria were isolated (54.9% of all strains), and their preponderance exhibited an upward trend from 2016 to 2019. This is consistent with previous studies conducted in India and other Asian countries, whereas Gram-positive bacteria were predominant in some studies conducted in the western countries (15, 18, 19). This difference may be associated with more recurrent diabetic foot and the inappropriate use of antibiotics in the developing countries. Lipsky et al. reported that Gram-positive bacteria were predominant in acute DFIs, whereas patients who had chronic wounds or had recently undergone antibiotic therapy were at an increased risk of infection with Gram-negative bacteria (20). With increasing Wagner's level, the pathogen spectrum gradually changed from Gram-positive bacteria to Gram-negative bacteria. In the present study, the species most frequently isolated from DFI patients was *P. aeruginosa* (19.6%). The findings of the present study are similar to those reported by Sugandhi et al. (21). *P. aeruginosa* is commonly resistant to antibiotics and can cause severe tissue damage for diabetic patients, resulting in sepsis and amputation (21–23). In the current study, 15% of the patients infected with *P. aeruginosa* ultimately underwent amputation, and 50% of all the patients who underwent amputation in the study had *P. aeruginosa* infections. One of the challenges in managing *P. aeruginosa* infections is an inherent resistance mechanism (17). In the present study, most of the patients infected with *P. aeruginosa* had a long history of diabetes, which may create conditions for opportunistic pathogen infection. From 2016 to 2019, the detection rate of fungi in DFI was only 2.0%. Notably, fungal infection is often secondary to the long-term use of a large number of antibacterial drugs. Fungal infections should not be ignored in clinical practice.

In the current study, *Staphylococcus* exhibited high susceptibility to vancomycin, imipenem, meropenem, and rifampicin. In previous studies, the proportions of methicillin-resistant *S. aureus* isolated from DFUs have ranged from 15 to 50% (21, 24, 25). The prevalence of methicillin-resistant *S. aureus* is increasing in clinical practice, and the so-called “super bacteria” have begun to emerge. Debridement should be performed in time to avoid secondary infection. It is also necessary to use glycopeptide cautiously to prevent the induction of new drug-resistant bacteria, and reasonable treatment measures should be utilized based on the results of drug susceptibility tests. In the present study, *Enterococcus* isolates were highly susceptible to vancomycin, meropenem, and rifampicin. This is consistent with the results of previous drug resistance studies in China (26, 27). *Acinetobacter* and *Pseudomonas* had the highest susceptibility to rifampicin, vancomycin, and meropenem. Rajalakshmi and Amsaveni (28) reported that imipenem was one of the most effective agents against Gram-negative bacteria including *P. aeruginosa*. In the current study, *Acinetobacter* and *Pseudomonas* exhibited low susceptibility to imipenem (38.1%) compared with that to vancomycin, meropenem, and rifampicin. In 2015, Perim et al. (29) reported that the rate of *Pseudomonas* resistance to imipenem reached 50%. Thus, the use of imipenem as the first-choice treatment for DFI with Gram-negative bacteria is no longer advisable.

The current study yielded several interesting results. Firstly, *Citrobacter* was predominant in female patients (80%). The reasons for this require further investigation. Secondly, *S. epidermidis* infections were the most difficult to cure. Up to 60% of the *S. epidermidis* infected patients in the study could not be cured. *S. epidermidis* infection is the most common DFI in patients aged > 70 years, with a DM history of >10 years and poor glycemic control. Lastly, *E. faecalis, E. coli*, and *S. aureus* are the top three pathogen strains associated with a foot injury, and these three strains are the most susceptible to therapy.

The fact that the efficacy of some antibiotics in the study decreased year by year warrants attention. The susceptibility of *Acinetobacter* and *Pseudomonas* to meropenem decreased from 100% in 2016 to 57.1% in 2019. Several antibiotics that were effective at the beginning of the study in 2016 were ineffective in subsequent years. Like *Klebsiella* and *Proteus*, which were once relatively susceptible to a wide range of antibiotics but no longer are, other species may now produce extended-spectrum beta-lactamases or carbapenemases, rendering them resistant to most of the commonly used drugs (30, 31). A major reason for the emergence of these resistant organisms may be inappropriate, typically unnecessary, and overly prolonged antibiotic treatment.

Previous studies have indicated a 33% prevalence of antibiotic resistant bacteria in DFI, and an increasing trend in recent years (32). In conjunction with the fact that DM patients are inherently susceptible to foot infections, it will be difficult to control DFIs effectively, which may lead to the expansion of infections. Therefore, microbiological examination and drug susceptibility testing prior to empirical antibiotic therapy were advised (33). There are surprisingly few published clinical trials on antibiotic therapy for DFIs. The data generated in the present study constitute contemporary observations. However, there are two limitations in our study. Firstly, only DFUs with dominant pathogen infection were included in the current study. Secondly, the administration of antibiotics was only based on the antibiotic susceptibility test of the dominant pathogen. In the future, polymicrobial detection will be performed to fully profile the pathogens of DFUs. Moreover, drug susceptibility tests will be applied to the major pathogens, and not only to the dominant ones. Furthermore, the combination use of antibiotics against the major pathogens will be considered.

## Data Availability Statement

The raw data supporting the conclusions of this article will be made available by the authors, without undue reservation.

## Ethics Statement

The studies involving human participants were reviewed and approved by the ethical committee of Tianjin Second Hospital. The patients/participants provided their written informed consent to participate in this study.

## Author Contributions

YWu and XL conceived and designed the study. WC and YWa collected data. YWa, HZ, SY, YL, and XL performed interpretation of data. WC, YWa, YWu, and XL wrote the manuscript. All authors read and approved the final manuscript.

## Funding

This research was funded by National Natural Science Foundation of China (NSFC) Grant 31701247 and Supporting Fund for Teacher's Research of Jining Medical University JYFC2018JS004 to XL.

## Conflict of Interest

The authors declare that the research was conducted in the absence of any commercial or financial relationships that could be construed as a potential conflict of interest.

## Publisher's Note

All claims expressed in this article are solely those of the authors and do not necessarily represent those of their affiliated organizations, or those of the publisher, the editors and the reviewers. Any product that may be evaluated in this article, or claim that may be made by its manufacturer, is not guaranteed or endorsed by the publisher.
