# Supplementary material for: The Profile of Microbiological Pathogens in Diabetic Foot Ulcers
Source: Front Med (Lausanne). 2021 Sep 21;8:656467. doi: 10.3389/fmed.2021.656467 (PMC8491778; doi:10.3389/fmed.2021.656467)
Supplement: Supplementary file 1 [file Table_1.DOCX]

Supplementary Material

**Table S1. The pathogen spectrum in each group**

| **Strain type** | **Gender** | | | | **age** | | | | | | | | **duration of type 2 DM (year)** | | | | | | | | **blood sugar control** | | | | **initial cause of infection** | | | | | |
| --- | --- | --- | --- | --- | --- | --- | --- | --- | --- | --- | --- | --- | --- | --- | --- | --- | --- | --- | --- | --- | --- | --- | --- | --- | --- | --- | --- | --- | --- | --- |
|  | **Female** | | **Male** | | **≤60** | | **61-70** | | **71-80** | | **≥81** | | **≤5** | | **6-10** | | **11-15** | | **≥16** | | **Poor** | | **well** | | **Injury** | | **Tinea pedis** | | **Ischemic rupture** | |
|  | **n** | **p (%)** | **n** | **p (%)** | **n** | **p (%)** | **n** | **p (%)** | **n** | **p (%)** | **n** | **p (%)** | **n** | **p (%)** | **n** | **p (%)** | **n** | **p (%)** | **n** | **p (%)** | **n** | **p (%)** | **n** | **p (%)** | **n** | **p (%)** | **n** | **p (%)** | **n** | **p (%)** |
| - ***gram-negative bacteria*** | **26** | **70.3** | **30** | **46.2** | **5** | **50.0** | **16** | **48.5** | **26** | **56.5** | **9** | **69.2** | **3** | **75.0** | **32** | **49.2** | **15** | **65.2** | **6** | **60.0** | **33** | **48.5** | **23** | **67.6** | **30** | **46.2** | **13** | **72.2** | **13** | **68.4** |
| *Pseudomonas aeruginosa* | 9 | 24.3 | 11 | 16.9 | 2 | 20.0 | 4 | 12.1 | 10 | 21.7 | 4 | 30.8 | 3 | 75.0 | 7 | 10.8 | 7 | 30.4 | 3 | 30.0 | 10 | 14.7 | 10 | 29.4 | 6 | 9.2 | 9 | 50.0 | 5 | 26.3 |
| *Klebsiella pneumoniae* | 2 | 5.4 | 6 | 9.2 | 0 | / | 3 | 9.1 | 3 | 6.5 | 2 | 15.4 | 0 | / | 5 | 7.7 | 1 | 4.3 | 2 | 20.0 | 4 | 5.9 | 4 | 11.8 | 4 | 6.2 | 4 | 22.2 | 0 | / |
| *Escherichia coli* | 5 | 13.5 | 10 | 15.4 | 3 | 30.0 | 7 | 21.2 | 4 | 8.7 | 1 | 7.7 | 0 | / | 12 | 18.5 | 3 | 13.0 | 0 | / | 10 | 14.7 | 5 | 14.7 | 14 | 21.5 | 0 | / | 1 | 5.3 |
| *Citrobacter* | 4 | 10.8 | 1 | 1.5 | 0 | / | 1 | 3.0 | 3 | 6.5 | 1 | 7.7 | 0 | / | 4 | 6.2 | 1 | 4.3 | 0 | / | 4 | 5.9 | 1 | 2.9 | 2 | 3.1 | 0 | / | 3 | 15.8 |
| *Morganella morganii* | 1 | 2.7 | 0 | / | 0 | / | 0 | / | 1 | 2.2 | 0 | / | 0 | / | 0 | / | 1 | 4.3 | 0 | / | 0 | / | 1 | 2.9 | 0 | / | 0 | / | 1 | / |
| *Enterobacter aerogenes* | 0 | / | 0 | / | 0 | / | 0 | / | 0 | / | 0 | / | 0 | / | 0 | / | 0 | / | 0 | / | 0 | / | 0 | / | 0 | / | 0 | / | 0 | / |
| *Acinetobacter lwoffii* | 0 | / | 0 | / | 0 | / | 0 | / | 0 | / | 0 | / | 0 | / | 0 | / | 0 | / | 0 | / | 0 | / | 0 | / | 0 | / | 0 | / | 0 | / |
| *Acinetobacter calcoaceticus* | 0 | / | 0 | / | 0 | / | 0 | / | 0 | / | 0 | / | 0 | / | 0 | / | 0 | / | 0 | / | 0 | / | 0 | / | 0 | / | 0 | / | 0 | / |
| *Enterobacter cloacae* | 5 | 13.5 | 2 | 3.1 | 0 | / | 1 | 3.0 | 5 | 10.9 | 1 | 7.7 | 0 | / | 4 | 6.2 | 2 | 8.7 | 1 | 10.0 | 5 | 7.4 | 2 | / | 4 | 6.2 | 0 | / | 3 | 15.8 |
| *others* | 0 | / | 0 | / | 0 | / | 0 | / | 0 | / | 0 | / | 0 | / | 0 | / | 0 | / | 0 | / | 0 | / | 0 | / | 0 | / | 0 | / | 0 | / |
| - ***gram-positive bacteria*** | **10** | **27.0** | **34** | **52.3** | **5** | **50.0** | **16** | **48.5** | **20** | **43.5** | **3** | **23.1** | **1** | **25.0** | **31** | **47.7** | **8** | **34.8** | **4** | **40.0** | **34** | **50.0** | **10** | **29.4** | **35** | **53.8** | **3** | **16.7** | **6** | **31.6** |
| *Enterococcus faecalis* | 2 | 5.4 | 10 | 15.4 | 1 | 10.0 | 2 | 6.1 | 8 | 17.4 | 1 | 7.7 | 0 |  | 10 | 15.4 | 1 | 4.3 | 1 | 10.0 | 11 | 16.2 | 1 | 2.9 | 10 | 15.4 | - | / | 2 | 10.5 |
| *Staphylococcus aureus* | 5 | 13.5 | 14 | 21.5 | 2 | 20.0 | 11 | 33.3 | 5 | 10.9 | 1 | 7.7 | 1 | 25.0 | 14 | 21.5 | 2 | 8.7 | 2 | 20.0 | 15 | 22.1 | 4 | 11.8 | 19 | 29.2 | - | / | 0 | / |
| *Streptococcus haemolyticus* | 1 | 2.7 | 7 | 10.8 | 2 | 20.0 | 2 | 6.1 | 4 | 8.7 | 0 |  | 0 | - | 6 | 9.2 | 2 | 8.7 | 0 | - | 4 | 5.9 | 4 | 11.8 | 4 | 6.2 | 2 | 11.1 | 2 | 10.5 |
| *Stap hylococcus epidermidis* | 2 | 5.4 | 3 | 4.6 | 0 | / | 1 | 3.0 | 3 | 6.5 | 1 | 7.7 | 0 | - | 1 | 1.5 | 3 | 13.0 | 1 | 10.0 | 4 | 5.9 | 1 | 2.9 | 2 | 3.1 | 1 | 5.6 | 2 | 10.5 |
| *others* | 0 | / | 0 | / | 0 | / | 0 | / | 0 | / | 0 |  | 0 | - | 0 |  | 0 | - | 0 | - | 0 | / | 0 | / | 0 | / | 0 | / | 0 | / |
| - ***fungal*** | **1** | **2.7** | **1** | **1.5** | **0** | **/** | **1** | **3.0** | **0** | **/** | **1** | **7.7** | **0** | **-** | **2** | **3.1** | **0** | **-** | **0** | **-** | **1** | **1.5** | **1** | **2.9** | **0** | **/** | **2** | **11.1** | **0** | **/** |
| *Candida albicans* | 1 | 2.7 | 1 | 1.5 | 0 | / | 1 | 3.0 | 0 | / | 1 | 7.7 | 0 | - | 2 | 3.1 | 0 | - | 0 | - | 1 | 1.5 | 1 | 2.9 | 0 | / | 2 | 11.1 | 0 | / |
| *Candida tropicalis* | 0 | / | 0 | / | 0 | / | 0 | / | 0 | / | 0 |  | 0 | - | 0 | - | 0 | - | 0 | - | 0 | / | 0 | / | 0 | / | 0 | / | 0 | / |
| *others* | 0 | / | 0 | / | 0 | / | 0 | / | 0 | / | 0 |  | 0 | - | 0 | - | 0 | - | 0 | - | 0 | / | 0 | / | 0 | / | 0 | / | 0 | / |
| **Ttotal** | **37** |  | **65** |  | **10** |  | **33** |  | **46** |  | **13** |  | **4** |  | **65** |  | **23** |  | **10** |  | **68** |  | **34** |  | **65** |  | **18** |  | **19** |  |

**Table S2. The pathogen distributions among groups**

| **Strain type** | **Gender** | | | | **age** | | | | | | | | **duration of type 2 DM (year)** | | | | | | | | **blood sugar control** | | | | **initial cause of infection** | | | | | |
| --- | --- | --- | --- | --- | --- | --- | --- | --- | --- | --- | --- | --- | --- | --- | --- | --- | --- | --- | --- | --- | --- | --- | --- | --- | --- | --- | --- | --- | --- | --- |
|  | **Female** | | **Male** | | **≤60** | | **61-70** | | **71-80** | | **≥81** | | **≤5** | | **6-10** | | **11-15** | | **≥16** | | **Poor** | | **well** | | **Injury** | | **Tinea pedis** | | **Ischemic rupture** | |
|  | **n** | **p (%)** | **n** | **p (%)** | **n** | **p (%)** | **n** | **p (%)** | **n** | **p (%)** | **n** | **p (%)** | **n** | **p (%)** | **n** | **p (%)** | **n** | **p (%)** | **n** | **p (%)** | **n** | **p (%)** | **n** | **p (%)** | **n** | **p (%)** | **n** | **p (%)** | **n** | **p (%)** |
| - ***gram negative bacteria*** | **26** | **46.4** | **30** | **53.6** | **5** | **8.9** | **16** | **28.6** | **26** | **46.4** | **9** | **16.1** | **3** | **7.1** | **32** | **57.1** | **15** | **26.8** | **6** | **10.7** | **33** | **58.9** | **23** | **41.1** | **30** | **53.6** | **13** | **23.2** | **13** | **23.2** |
| *Pseudomonas aeruginosa* | 9 | 45.0 | 11 | 55.0 | 2 | 10.0 | 4 | 20.0 | 10 | 50.0 | 4 | 20.0 | 3 | 15.0 | 7 | 35.0 | 7 | 35.0 | 3 | 15.0 | 10 | 50.0 | 10 | 50.0 | 6 | 30.0 | 9 | 45.0 | 5 | 25.0 |
| *Klebsiella pneumoniae* | 2 | 25.0 | 6 | 75.0 | 0 | / | 3 | 37.5 | 3 | 37.5 | 2 | 25.0 | 0 | / | 5 | 62.5 | 1 | 12.5 | 2 | 25 | 4 | 50.0 | 4 | 50.0 | 4 | 50.0 | 4 | 50.0 | 0 | / |
| *Escherichia coli* | 5 | 33.3 | 10 | 66.7 | 3 | 20.0 | 7 | 46.7 | 4 | 26.7 | 1 | 6.7 | 0 | / | 12 | 80.0 | 3 | 20.0 | 0 | / | 10 | 66.7 | 5 | 33.3 | 14 | 93.3 | 0 | / | 1 | 6.7 |
| *Citrobacter* | 4 | 80 | 1 | 20 | 0 | / | 1 | 20.0 | 3 | 60.0 | 1 | 20.0 | 0 | / | 4 | 80.0 | 1 | 20.0 | 0 | / | 4 | 80.0 | 1 | 20.0 | 2 | 40.0 | 0 | / | 3 | 60.0 |
| *Morganella morganii* | 1 | 100 | 0 | / | 0 | / | 0 | / | 1 | 100 | 0 | / | 0 | / | 0 | / | 1 | 100 | 0 | / | 0 | / | 1 | 100 | 0 | / | 0 | / | 1 | 100 |
| *Enterobacter cloacae* | 5 | 71.4 | 2 | 28.6 | 0 | / | 1 | 14.3 | 5 | 71.4 | 1 | 14.3 | 0 | / | 4 | 57.1 | 2 | 28.6 | 1 | 14.3 | 5 | 71.4 | 2 | 28.6 | 4 | 57.1 | 0 | / | 3 | 42.9 |
| - ***gram-positive bacteria*** | **10** | **22.7** | **34** | **77.3** | **5** | **11.4** | **16** | **36.4** | **20** | **45.5** | **3** | **6.8** | **1** | **2.3** | **31** | **70.5** | **8** | **18.2** | **4** | **9.1** | **34** | **77.3** | **10** | **22.7** | **35** | **79.5** | **3** | **6.8** | **6** | **13.6** |
| *Enterococcus faecalis* | 2 | 16.7 | 10 | 83.3 | 1 | 8.3 | 2 | 16.7 | 8 | 66.7 | 1 | 8.3 | 0 | / | 10 | 83.3 | 1 | 8.3 | 1 | 8.3 | 11 | 91.7 | 1 | 8.3 | 10 | 83.3 | 0 | / | 2 | 16.7 |
| *Staphylococcus aureus* | 5 | 26.3 | 14 | 73.7 | 2 | 10.5 | 11 | 57.9 | 5 | 26.3 | 1 | 5.3 | 1 | 5.3 | 14 | 73.7 | 2 | 10.5 | 2 | 10.5 | 15 | 78.9 | 4 | 21.1 | 19 | 100 | 0 | / | 0 | / |
| *Streptococcus haemolyticus* | 1 | 12.5 | 7 | 87.5 | 2 | 25.0 | 2 | 25.0 | 4 | 50.0 | 0 | / | 0 | / | 6 | 75.0 | 2 | 25.0 | 0 | / | 4 | 50.0 | 4 | 50.0 | 4 | 50.0 | 2 | 25.0 | 2 | 25.0 |
| *Stap hylococcus epidermidis* | 2 | 40.0 | 3 | 60.0 | 0 | / | 1 | 20.0 | 3 | 60.0 | 1 | 20.0 | 0 | / | 1 | 20.0 | 3 | 60.0 | 1 | 20.0 | 4 | 80.0 | 1 | 20.0 | 2 | 40.0 | 1 | 20.0 | 2 | 40.0 |
| - ***fungal*** | **1** | **50** | **1** | **50** | **0** | **/** | **1** | **50.0** | **0** | **/** | **1** | **50.0** | **0** | **/** | **2** | **100** | **0** | **/** | **0** | **/** | **1** | **50.0** | **1** | **50.0** | **0** | **/** | **2** | **100** | **0** | **/** |
| *Candida albicans* | 1 | 50 | 1 | 50 | 0 | / | 1 | 50.0 | 0 | / | 1 | 50.0 | 0 | / | 2 | 100 | 0 | / | 0 | / | 1 | 50.0 | 1 | 50.0 | 0 | / | 2 | 100 | 0 | / |
| **Ttotal** | **37** | **36.3** | **65** | **63.7** | **10** | **9.8** | **33** | **32.4** | **46** | **45.1** | **13** | **12.7** | **4** | **3.9** | **65** | **63.7** | **23** | **22.5** | **10** | **9.8** | **68** | **66.7** | **34** | **33.3** | **65** | **63.7** | **18** | **17.6** | **19** | **18.6** |
